# Supplementary material for: WordCommentsAnalyzer: A windows software tool for qualitative research
Source: F1000Res. 2018 Sep 4;7:536. Originally published 2018 May 3. [Version 2] doi: 10.12688/f1000research.14819.2 (PMC6137411; doi:10.12688/f1000research.14819.2)
Supplement: Supplementary file 3 [file f1000research-7-17614-s0002.tgz › b2a79496-4ada-47c4-9682-a99ce867836d.zip!/Eight Iranian Health Journals/Salmand_Iranian Journal of Ageing _10.docx]

© Salmand_Iranian Journal of Ageing . This work is licensed under CC BY-NC 4.0 (https://creativecommons.org/licenses/by-nc/4.0/).

Volume & Issue: دوره 10 - شماره 4

Title: ارزیابی ارتباط افسردگی و میزان سوءرفتار در سالمندان شهر کاشان

http://salmandj.uswr.ac.ir/article-1-903-fa.html

اهداف: سوءرفتار با سالمند یکی از مشکلات روانی و اجتماعی جوامع است که تاثیر بسزایی در کاهش سلامت روانی سالمندان دارد و می‌تواند به‌عنوان یکی از عوامل زمینه‌ساز ایجاد افسردگی سالمندی باشد. هدف این مطالعه ارزیابی ارتباط بین افسردگی سالمندی و سوءرفتار در سالمندان شهر کاشان در سال 1393 بود. مواد و روش‌ها: مطالعه حاضر پژوهشی توصیفی-مقطعی بود که در سال 1393 انجام شد. جامعه پژوهش 500 نفر از سالمندان بالای 60 سال شهر کاشان بودند. نمونه‌گیری به‌صورت تصادفی انجام شد که از بین 10 مرکز بهداشتی درمانی شهر کاشان انتخاب شد. ابزار مطالعه شامل اطلاعات جمعیت‌شناختی و پرسش‌نامه سوءرفتار با سالمند در خانواده (0/975=α) و پرسشنامه ارزیابی افسردگی سالمندی (0/90=α) بود. یافته‌ها: نتایج نشان داد 400 نفر (80درصد) از شرکت‌کننده حداقل یکی از انواع سوءرفتار را در یک سال گذشته تجربه کرده بودند. بیشترین میزان شیوع سوءرفتار مربوط به سوءرفتار مالی (45/6درصد) بود و 79/2درصد، درجات مختلفی از افسردگی داشتند. نتایج رگرسیون خطی نشان داد غفلت عاطفی (0/001=P و 0/20=Beta), غفلت مالی (0/036=P و 0/117=Beta) و طردشدگی (0/001=P و 0/241=Beta) با افسردگی ارتباط معنادار داشت. براساس آزمون‌ها، ارتباط معناداری بین نمره سوءرفتار با افسردگی (0/001=P و 0/490=r) وجود داشت. نتیجه‌گیری: نتایج این مطالعه بیانگر اهمیت و ضرورت ارائه خدمات بهداشتی-مراقبتی و حمایتی برای شناسایی سالمندان افسرده قربانی سوءرفتار و پیشگیری به‌منظور حل این معضل و بهبود وضعیت آنها بود.

Title: پایایی آزمون-بازآزمون، روایی سازه و همخوانی درونی نسخه فارسی‌شده آزمون «مقیاس تعادل پیشرفته فولرتون» در سالمندان ایرانی مقیم جامعه

http://salmandj.uswr.ac.ir/article-1-844-fa.html

اهداف: هدف این مطالعه بررسی روایی سازه، پایایی آزمون-بازآزمون و همخوانی درونی نسخه فارسی‌شده «مقیاس تعادل پیشرفته فولرتون»، برای ارتقای کیفیت ارزیابی تعادل در گروه سالمندان مقیم جامعه در ایران است. مواد و روش‌ها: مقیاس تعادل پیشرفته فولرتون به روش پیش‌رو-پس‌رو به زبان فارسی ترجمه شد. سپس با نمونه‌گیری به روش غیراحتمالی ساده بر مبنای معیارهای ورود به مطالعه، 88 سالمند با عملکرد بالا (با میانگین سنی 67/15، انحراف معیار 5/79 و دامنه 26) به مطالعه وارد شدند. آزمون فارسی شده «تعادل پیشرفته فولرتون» دو بار با فاصله زمانی 4-2 روز توسط یک آزمونگر (کاردرمان) روی هر فرد اجرا شد. همچنین از آزمون‌های تعادلی بِرگ، زمان خیزش و رفت و آزمون رسیدن عملکردی برای بررسی روایی سازه استفاده گردید. یافته‌ها: میزان توافق آزمون-بازآزمون برای ‌موارد آزمون تعادلی فولرتون خوب تا بسیارخوب (Kappa=0/63-1) محاسبه شد. ضریب تکرارپذیری نسبی بسیار مطلوب (0/89=CCI)، شاخص تکرارپذیری مطلق مطلوب (0/17=SEM) و کمترین تغییرات مشهود 0/46 به‌دست آمد. در روایی سازه همبستگی مطلوبی بین آزمون فولرتون با آزمون بِرگ (0/65=P)، با آزمون رسیدن عملکردی (0/62=г) و زمان خیزش و رفت (0/77-=г) به‌دست آمد. در هر دو مرحله اول و دوم ارزیابی، میزان همخوانی درونی قابل قبول (0/84-0/83=α) بود. نتیجه‌گیری: نسخه فارسی آزمون تعادل پیشرفته فولرتون، یک ابزار روان‌سنجی بسیار مطلوب برای ارزیابی ابعاد چندگانه تعادل در سالمندان ایرانی مقیم جامعه است.

Title: ارتباط راهبردهای مقابله‌ای و مذهب‌گرایی با رضایت‌مندی زندگی سالمندان ساکن در سرای سالمندان اهواز

http://salmandj.uswr.ac.ir/article-1-787-fa.html

اهداف: این مطالعه با هدف بررسی ارتباط راهبردهای مقابله‌ای و مذهب‌گرایی با رضایت‌مندی زندگی در سالمندان ساکن در سرای سالمندی اهواز انجام شد . مواد و روش‌ها: مطالعه حاضر یک مطالعه توصیفی از نوع همبستگی است که در سال 1392 روی 100 نفر سالمند (64 مرد و 36 زن) ساکن در خانه‌های سالمندی در شهرستان اهواز انجام شد. برای جمع‌آوری اطلاعات از سه پرسش‌نامه راهبردهای مقابله با موقعیت‌های تنش‌زا، جهت‌گیری مذهبی آلپورت و راس و مقیاس رضایت از زندگی استفاده شد که دارای روایی و پایایی مناسبی است. معیارهای ورود به این مطالعه عبارت بود از: درک زبان فارسی، نداشتن دمانس، تمایل به شرکت در پژوهش و دارابودن حداقل 60 سال سن یا بالاتر. معیارهای خروج از این مطالعه را داشتن اختلالات جسمانی یا روان‌پزشکی که منجر به افسردگی شدید یا اضطراب در سالمند شود، دمانس و عدم درک زبان فارسی تشکیل می‌داد. برای تحلیل داده‌ها از نسخه 19 نرم‌افزار SPSS، آزمون‌های آماری تحلیل واریانس یک‌راهه، رگرسیون چندمتغیره و ضریب همبستگی پیرسون استفاده شد.  یافته‌ها: بررسی‌های این مطالعه نشان داد که بین مذهب‌گرایی و راهبردهای مقابله‌ای با رضایت‌مندی زندگی سالمندان ارتباط معنی‌داری وجود دارد (0/005=P). نتیجه‌گیری: راهبردهای مقابله‌ای و مذهب‌گرایی به‌طور معنی‌داری قادر است، رضایت‌مندی زندگی سالمندان را پیش‌بینی کند. یافته‌های این پژوهش می‌تواند به مسئولین خانه سالمندان کمک نماید تا با تقویت آموزه‌های مذهبی و ارائه راهبردهای مقابله‌ای رضایت‌مندی از زندگی را در سالمندان افزایش دهند و از طریق راهنمایی سالمندان در انتخاب راهکارهای مؤثرتر برای تطابق با عوامل استرس‌زا و مشکلات همراه کهولت سن، به رضایت از زندگی این افراد کمک نمایند.

Title: بررسی و مقایسه سلامت روانی و شادکامی سالمندان فعال از نظر حرکتی، طبیعت گرد و غیر فعال در شهرستان ایلام

http://salmandj.uswr.ac.ir/article-1-781-fa.html

اهداف: هدف تحقیق حاضر بررسی و مقایسه سلامت روانی و شادکامی سالمندان فعال از نظر حرکتی، طبیعت‌گرد و غیرفعال در شهرستان ایلام است. مواد و روش‌ها: در این پژوهش، 514 نفر از سالمندان (152 فعال، 229 غیرفعال و 133 طبیعت‌گرد) انتخاب شدند. ابزار تحقیق، پرسش‌نامه‌های  سلامت روان (28-GHQ) و شادکامی آرجیل بود. برای بررسی نتایج تحقیق از روش‌های آماری تحلیل واریانس یک‌راهه و در صورت معناداری، از آزمون تعقیبی توکی استفاده گردید. یافته‌ها: نتایج تحقیق نشان می‌دهد که در مقیاس سلامت روانی بین تمامی گروه‌ها تفاوت معناداری وجود دارد، اما در مقیاس شادکامی بین گروه طبیعت‌گرد با گروه‌های فعال و غیرفعال تفاوت معنادار و بین فعال و غیرفعال تفاوت معناداری وجود ندارد. نتیجه‌گیری: شرکت در فعالیت‌های ورزشی و تفریحی مانند طبیعت‌گردی به‌همراه دوستان یا اعضای خانواد‌ه یکی از مناسب‌ترین فعالیت‌های پیشنهادی برای سالمندان است که منجر به سلامت روان و شادکامی آنان می‌گردد.

Title: سوء‌رفتار خانگی با سالمندان روستایی شهرستان دزفول و ارتباط آن با کیفیت زندگی آنان

http://salmandj.uswr.ac.ir/article-1-888-fa.html

اهداف: سالمندان ازجمله گروه‌های آسیب‌پذیر جامعه هستند و پدیده سالمندآزاری ازجمله چالش‌های مطرح در حیطه مراقبت و حمایت از سالمندان است. پژوهش حاضر با هدف تعیین سوء‌رفتار خانگی با سالمندان روستایی شهرستان دزفول و ارتباط آن با کیفیت زندگی آنان در سال 1392صورت گرفت. مواد و روش‌ها: پژوهش حاضر مطالعه‌ای مقطعی از نوع همبستگی بود که نمونه‌گیری آن به‌شکل خوشه‌ای تصادفی انجام گرفت و در آن 210 سالمند 60 سال و بالاتر ساکن روستاهای شهر دزفول شرکت کردند. سالمندانی که براساس نسخه فارسی آزمون کوتاه وضعیت شناختی فاقد اختلال شناختی بودند، پس از کسب رضایت آگاهانه وارد مطالعه شدند. سپس پرسش‌نامه بررسی سوءرفتار خانواده نسبت به سالمندان، نسخه فارسی پرسش‌نامه SF-36 به‌منظور سنجش کیفیت زندگی از منظر سلامت و نیز پرسش‌نامه جمع‌آوری اطلاعات دموگرافیک و فردی برای آنان تکمیل گردید. داده‌ها پس از جمع‌آوری با به‌کارگیری آمار توصیفی و آزمون‌های مجذور کای و تی مستقل تجزیه و تحلیل شدند. یافته‌ها: یافته‌های پژهش نشان داد که 60/5درصد از نمونه‌های پژوهش، حداقل یکی از انواع سوءرفتار را تجربه کرده بودند.بیشترین میزان شیوع سوءرفتار مربوط به غفلت عاطفی (38/6درصد) و کمترین آن مربوط به غفلت مراقبتی (7/3درصد) بود.در این مطالعه، گروه سالمندان بدون تجربه سوءرفتار، نسبت به سالمندان باتجربه سوءرفتار در کلیه ابعاد هشت‌گانه کیفیت زندگی به‌جز 2 بُعد عملکرد جسمانی و سلامت عمومی، به‌طور معنی‌داری از کیفیت زندگی بهتری برخوردار بودند (0/05>P). نتیجه‌گیری: با وجود تأکید زیادی که در فرهنگ ما برای احترام به سالمند وجود دارد، سالمندان انواع مختلف سوءرفتار را تجربه می‌کنند؛ بنابراین، توجه ویژه و ایجاد حساسیت در افراد و سازمان‌های مرتبط با این پدیده به‌منظور پیشگیری از نتایج بلندمدت آن موردنیاز است.

Title: ارتباط بین فعالیت فیزیکی و کنترل تعادل در سالمندان

http://salmandj.uswr.ac.ir/article-1-800-fa.html

اهداف: سالمندی فرایندی است که منجر به کاهش کیفیت زندگی و ایجاد وابستگی می‌شود. سالمندی بخش مهمی از جامعه بشری است و تغییرات ایجاد شده بر شیوه زندگی سالمند اطرافیانش و کل جامعه تأثیر می‌گذارد. با توجه به اهمیت این موضوع و ازآنجاکه مطالعات اندکی در این زمینه انجام شده است، هدف از این مطالعه، بررسی فعالیت فیزیکی و کنترل تعادل در سالمندان و ارتباط بین آنها بوده است. مواد و روش‌ها: در این مطالعه مقطعی، 1614 سالمند شهر امیرکلا طی سال‌های 91-90 شرکت داشتند. میزان فعالیت فیزیکی با استفاده از پرسش‌نامه استاندارد فعالیت بدنی در سالمندان و کنترل تعادل با آزمون تعادل برگ اندازه‌گیری شد. سپس اطلاعات با استفاده از آزمون‌های آماری کای-دو و تی در نرم‌افزار SPSS در سطح 0/05 مورد تجزیه‌وتحلیل قرار گرفت. یافته‌ها: در این مطالعه 1614 سالمند مورد بررسی قرار گرفتند که از این تعداد 883 نفر مرد و 733 نفر زن بودند. میانگین سنی مردان 7/68±69/96 و میانگین سنی زنان 7/02±68/66 بوده است. ارتباط بین میزان فعالیت فیزیکی و کنترل تعادل در افراد مورد مطالعه از نظر آماری معنادار و مستقیم بوده است (0/000=P). نتیجه‌گیری: نتایج این مطالعه بیانگر وجود ارتباط معنادار بین فعالیت فیزیکی و کنترل تعادل در سالمندان است. نتیجه حاصل از این مطالعه این موضوع را تداعی می‌کند که می‌توان با برنامه‌ریزی برای افزایش فعالیت فیزیکی، به‌میزان زیادی موجب بهبود کنترل تعادل در سالمندان شد و از مشکلات بعدی مانند زمین‌خوردن کاست.

Title: بررسی تأثیر الگوی توانمندسازی خودمدیریتی بر کیفیت زندگی سالمندان مبتلا به پرفشاری خون

http://salmandj.uswr.ac.ir/article-1-796-fa.html

اهداف: پرفشاری خون با شیوه زندگی و کیفیت زندگی ارتباط تنگاتنگی دارد و در صورت کنترل‌نکردن باعث ایجاد ناتوانی و کاهش سطح کیفیت زندگی مبتلایان می‌شود. مطالعه حاضر با هدف تأثیر الگوی توانمندسازی خودمدیریتی بر کیفیت زندگی سالمندان مبتلا به پرفشاری خون اجرا گردید.  مواد و روش‌ها: پژوهش حاضر، مطالعه‌ای کارآزمایی بالینی است که روی سالمندان مبتلا به پرفشاری خون شهر بوشهر اجرا گردید. نمونه‌گیری به‌صورت مبتنی بر هدف بود، اما نمونه‌ها به‌صورت تصادفی به دو گروه آزمون یا کنترل تخصیص داده شدند. مداخله در 5 مرحله (خودآگاهی از سطح عملکرد و انتظارات از خود، هدف‌گذاری مطلوب توسط مددجو، برنامه‌ریزی، اصلاح ساختار و ارزشیابی) براساس الگوی توانمندسازی خودمدیریتی صورت گرفت. اطلاعات از طریق پرسشنامه‌های اطلاعات دموگرافیک و کیفیت زندگی جمع‌آوری و با نسخه 18 نرم‌افزار SPSS در سطح معناداری 0/05 تجزیه‌وتحلیل گردید. یافته‌ها: با به‌کارگیری برنامه توانمندسازی سالمندان، میانگین نمره کل کیفیت زندگی و ابعاد آن در گروه آزمون بعد از مداخله نسبت به قبل از مداخله تفاوت معناداری را نشان داد (0/05>P). به‌جز بُعد محدودیت فعالیت که بعد از مداخله در این بُعد افزایش نمره مشاهده شد، تفاوت معنادار آماری مشاهده نشد. نمره کل سطح کیفیت زندگی و دیگر ابعاد آن در گروه کنترل بعد از مداخله نسبت به قبل از مداخله تغییری نداشت و از نظر آماری تفاوت معناداری مشاهده نگردید (0/05). اختلاف نمره کل کیفیت زندگی قبل و بعد از مداخله در گروه آزمون نیز نسبت به اختلاف نمره در گروه کنترل تفاوت معناداری را نشان داد (P<0/05) نتیجه‌گیری: اجرای اصولی الگوی توانمندسازی خودمدیریتی با تأکید بر سالمندان مبتلا به پرفشاری توانست به بهبود و ارتقای کیفیت زندگی بینجامد؛ در نتیجه، این رویکرد می‌تواند در برنامه­‌های مراقبتی راهکاری مناسب باشد و کیفیت زندگی را در دوران سالمندی بهبود  بخشد.

Title: رابطه ساختار قدرت خانواده، تعارضات زناشویی و سلامت روان در مردان بازنشسته اداره آموزش‌و‌پرورش استان البرز

http://salmandj.uswr.ac.ir/article-1-703-fa.html

اهداف: هدف از انجام این پژوهش بررسی رابطه بین ساختار قدرت خانواده، تعارضات زناشویی و خرده‌مقیاس‌های آنان با سلامت روان در مردان بازنشسته اداره آموزش‌وپرورش استان البرز بود. مواد و روش‌ها: در این پژوهش، 341 نفر از مردان بازنشسته آموزش‌و‌پرورش استان البرز به روش نمونه‌گیری غیرتصادفی انتخاب و پرسشنامه‌های سلامت روان (28-GHQ)، تعارضات زناشویی و ساختار قدرت خانواده در میان آنان به‌اجرا درآمد. اطلاعات کسب‌شده با استفاده از روش‌های آماری ضریب آلفای کرونباخ، ضریب همبستگی پیرسون و رگرسیون گام‌به‌گام مورد تجزیه‌وتحلیل قرار گرفت. یافته‌ها: نتایج نشان داد بین ساختار قدرت خانواده (r=-0/527 و P=0/000)، قلمرو قدرت در خانواده (r=-0/437 و P=0/000)، ساخت قدرت خانواده (r=-0/484 و P=0/000)، شیوه اعمال قدرت زن و شوهر (r=-0/414 و P=0/000)، اتحاد و ائتلاف (r=-0/411 و P=0/000) و رهبری (r=-0/479 و P=0/000) با سلامت روان، رابطه منفی و معناداری وجود دارد. بین تعارضات زناشویی (r=0/56 و P=0/000)، افزایش جلب حمایت فرزند (r=0/46 و P=0/000)، افزایش رابطه با خویشاوندان خود (r=0/435 و P=0/000)، کاهش رابطه با خویشاوندان همسر (r=0/432 و P=0/000)، جداکردن امور مالی (r=0/448 و P=0/000)، کاهش همکاری زوجین (r=0/432 و P=0/000)، افزایش واکنش‌های هیجانی به یکدیگر (r=0/564 و P=0/000)، کاهش رابطه جنسی (r=0/372 و P=0/000)، کاهش ارتباط مؤثر (r=0/506 و P=0/000) با سلامت روان، رابطه مثبت و معناداری وجود دارد. افزایش واکنش‌های هیجانی (P=0/000)، ساخت قدرت خانواده (P=0/000)، قلمرو قدرت در خانواده (P=0/000)، کاهش رابطه جنسی (P=0/008)، کاهش ارتباط مؤثر (P=0/000)، اتحاد و ائتلاف (P=0/014) و کاهش رابطه با خویشاوندان همسر و دوستان (P=0/022) به‌خوبی می‌توانند سلامت روان مردان بازنشسته را پیش‌بینی کنند. نتیجه‌گیری: نتایج پژوهش حاکی از آن است که ساختار قدرت خانواده و تعارضات زناشویی بر سلامت روان مردان بازنشسته مؤثر است و ساختار قدرت نامساوی‌تر و تعارضات زناشویی بیشتر منجر به کاهش سلامت روان مردان بازنشسته می‌شود.

Title: بررسی رویکردی مؤثر به پیری: مطالعه نظریه داده‌بنیاد

http://salmandj.uswr.ac.ir/article-1-935-fa.html

اهداف: با توجه به آسیب‌پذیری تعداد قابل ملاحظه‌ای از سالمندان به‌علت مسائل گوناگون جسمانی، روانی-اجتماعی و خانوادگی، هدف پژوهش حاضر ارائه یک نظریه براساس گردآوری نظام‌مند داده‌ها در حوزه سالمندی است. مواد و روش‌ها: روش مطالعه‌ حاضر نوع خاصی از سنت پژوهش کیفی به نام نظریه داده‌بنیاد است و نمونه پژوهش شامل 20 نفر از سالمندان (10 زن و 10 مرد) بود که به‌شیوه‌ نمونه‌گیری هدفمند انتخاب شدند. جامعه آماری پژوهش حاضر را سطح جامعه شامل مکان‌های عمومی مانند پارک‌ها، مکان‌های تفریحی، سالن‌های انتظار ادارات و بیمارستان‌ها تشکیل می‌داد. ضمن انجام مصاحبه نیمه‌ساختاریافته با 20 نفر از سالمندان شهر تبریز و بررسی نظرات اعضای خانواده آنها، مجموعه‌ای از مضامین اولیه طی فرایند کدگذاری بازگردآوری و از بطن آن‌ها مقوله‌هایی استخراج گردید. سپس در مرحله کدگذاری محوری، پیوند میان این مقوله‌ها ذیل عناوین شرایط علّی، پدیده محوری، مقوله راهبردی، شرایط محیطی، مقوله واسطه‌ای و مقوله پیامدی در قالب پارادایم تعیین شد. در ادامه و در مرحله کدگذاری گزینشی، اجزای پارادایم کدگذاری تشریح، سیر سالمندی ترسیم و نظریه خلق شد. یافته‌ها: نتایج پژوهش، 6 مؤلفه اصلی و 39 زیرمؤلفه را برای پیری استخراج و آن‌ها را در قالب الگوی نظری بر مبنای نظریه داده‌بنیاد ارائه نموده است. نتیجه‌گیری: یافته‌های پژوهش ضمن اشاره به نیازهای بهداشت روانی و مسائل خانوادگی، هیجانی سالمندان، به شناخت و مفهوم‌پردازی متغیرهای مختلف مؤثر بر زندگی سالمندان براساس نظریه داده بنیاد تأکید می‌کند.

Title: بررسی اثربخشی مداخله مددکاری اجتماعی بر کاهش آزار سالمندان

http://salmandj.uswr.ac.ir/article-1-1017-fa.html

اهداف: افزایش آزار سالمندان در کشورهای در‌حال‌توسعه از جمله ایران، به مشکلی بهداشتی تبدیل خواهد شد. هدف از این پژوهش، بررسی تأثیر مداخله مددکاری اجتماعی با رویکرد شناختی-رفتاری بر کاهش تجربه آزار سالمندان بود. مواد و روش‌ها: 29 سالمند در دو گروه مداخله (14=n) و کنترل (15=n) شرکت کردند. سالمندان و خانواده در قالب یک کارآزمایی بالینی از شهریورماه تا دی‌ماه 1393 وارد پژوهش شدند و به‌صورت تصادفی ساده در دو گروه مداخله و کنترل قرار گرفتند. گروه مداخله، مداخله 5 جلسه‌ای مددکاری اجتماعی را با رویکرد شناختی-رفتاری مبتنی‌بر خانواده دریافت کردند. پرسش‌نامه «سوءرفتار نسبت به سالمندان در خانواده» برای ارزیابی سالمندآزاری در پیش‌آزمون، پس‌آزمون و پیگیری به‌کارگرفته رفت. از تحلیل واریانس با اندازه‌های مکرر و آزمون ویل‌کاکسون برای تحلیل داده‌ها استفاده شد. یافته‌ها: همه شرکت‌کنندگان، تجربه حداقل یک نوع آزار را گزارش کردند و 75/9درصد آنها فرزندان خود را به‌عنوان «آزارگر» معرفی کردند. تحلیل واریانس با اندازه‌های مکرر نشان داد که مددکاری اجتماعی شناختی-رفتاری مبتنی‌بر خانواده در کاهش نمره کل سالمندآزاری مؤثر بوده است. آزمون ویل‌کاکسون کاهش معناداری را در خرده‌مقیاس‌های غفلت مراقبتی (0/01=P)، سوءرفتار روان‌شناختی (0/003=P)، سوءرفتار مالی (0/01=P)، سلب اختیار (0/005=P) و غفلت مالی (0/02=P) گروه مداخله نشان داد. درحالی که مداخله در مورد سوءرفتار جسمی (0/31=P) اثربخش نبوده است. نتیجه‌گیری: نتایج این پژوهش نشان داد که مددکاری اجتماعی شناختی-رفتاری مبتنی بر خانواده بر کاهش آزار سالمندان مؤثر است. برای پژوهش‌های بعدی پیشنهاد می‌شود پژوهش مشابه با نمونه بزرگ‌تر و پیگیری 3 و 6 ماهه انجام گیرد.

Title: تبیین چگونگی سبک زندگی سالمندان: رویکرد نظریه زمینه‌ای

http://salmandj.uswr.ac.ir/article-1-741-fa.html

اهداف: جامعه ما که به سالمندی گام نهاده، از نظر مراقبت بهداشتی با مسائل گوناگونی روبه‌رو است. شواهد موجود نشان می‌دهد که علت بسیاری از مرگ‌ومیرها نتیجه سبک زندگی ناسالم است و این مهم با افزایش سن و کهولت، نمود بیشتری پیدا می‌کند و باعث مشکلات بسیاری در دوره سالمندی می‌شود. به‌دلیل اهمیت پیشگیری از عوامل خطر و همچنین تداوم مراقبت از سالمند و با توجه به اینکه در زمینه سبک زندگی سالمندان در ایران به روش کیفی کمتر پرداخته شده است، پژوهش حاضر با هدف تبیین مفهوم، ساختار و چگونگی و فرایند اتخاذ سبک زندگی سالمندان انجام شد. مواد و روش‌ها: برای دستیابی به هدف این مطالعه، مطالعه‌ای کیفی با رویکرد نظریه زمینه‌ای انجام شد. در این مطالعه تبیین مفهوم، ساختار و چگونگی سبک زندگی در متن و بستر زندگی خود سالمند با استفاده از نمونه‌گیری به‌صورت مبتنی‌بر هدف در آغاز و تا رسیدن به اشباع اطلاعات، به‌صورت نمونه‌گیری نظری ادامه یافت. ابزار اصلی جمع‌آوری داده مصاحبه نیمه‌ساختار بود. مشارکت‌کنندگان این مطالعه سالمندان (که همراه خانواده زندگی می‌کردند)، بودند. در مجموع با 23 مشارکت‌کننده مرد و زن، 29 مصاحبه عمیق انفرادی انجام و مصاحبه‌ها با روش استراوس و کوربین 1998 تجزیه‌وتحلیل شد. یافته‌ها: از تجزیه و تحلیل داده‌ها هشت مقوله محوری به‌دست آمد: محدودیت‌های سالمندی، رفتارهای روزمره، ساختار خانوادگی-اجتماعی نامطلوب، خودمدیریتی، نقصان سلامتی، کارآمدی ناکافی، آمادگی ناکافی و تقیّد به معنویات. مقوله رفتارهای روزمره بیانگر پدیده، مقوله خودمدیریتی بیانگر راهبردهای اتخاذشده توسط افراد و مقوله نقصان سلامتی پیامد این راهبردها بود. سه مقوله تقیّد به معنویات، کارآمدی ناکافی و آمادگی ناکافی تاثیرگذار بر خودمدیریتی بود؛ یعنی راهبرد را تحت‌تأثیر قرار داده بودند. نتیجه‌گیری: با توجه به پیامدهای حاصل از راهبردهای اتخاذ شده و براساس تعاریف سلامتی، می‌توان چنین استدلال نمود که سالمندان از سبک زندگی کامل برخوردار نیستند و سلامتی آنان دچار نقصان شده است. با عنایت به نتایج نظریه زمینه‌ای، اصلاح و تقویت رفتارهای سبک زندگی از ضروریات به‌شمار می‌آید.

Title: ادراک سالمندان از شیوه زندگی سالم: مطالعه‌ای کیفی

http://salmandj.uswr.ac.ir/article-1-914-fa.html

اهداف: شیوه زندگی سالم به‌عنوان یکی از عوامل اجتماعی موثر بر سلامت، منبع مهمی برای ارتقای سلامت سالمندان به‌شمار می‌آید. تبیین و شناخت مفهوم شیوه زندگی سالم با توجه به وابستگی آن به ساختارها و عوامل اجتماعی، می‌تواند بر مناسب‌سازی مداخلات ارتقای سلامت در جوامع مختلف تأثیر قابل توجهی گذارد. هدف این مطالعه، تحلیل تجارب و ادراکات سالمندان از شیوه زندگی سالم بود. مواد و روش‌ها: این مطالعه با رویکرد تحلیل محتوایی عرفی صورت گرفت. نمونه 16 نفره مبتنی‌بر هدف، از میان سالمندان مقیم جامعه شهر تهران که بالاتر از 60 سال عمر داشتند، انتخاب شدند. داده‌ها به روش مصاحبه عمیق جمع‌آوری و تحلیل شد. برای کسب اطمینان‌پذیری، از راهبردهای درگیری طولانی‌مدت مرتبط با موضوع مطالعه، حساب‌رسی مراحل تحلیل توسط پژوهشگر همکار، تأیید یافته‌ها توسط شرکت‌کنندگان و شرح مفصّل فرایند مطالعه استفاده شد. یافته‌ها: یافته‌ها نشانگر دو طبقه اصلی رفتارهای ارتقادهنده سلامت سالمند متمرکز بر فرد و رفتارهای متمرکز بر جامعه بود که در قالب 12 مضمون عادات بهداشتی، ایفای نقش‌های اجتماعی، داشتن ارتباط با دیگران، داشتن خواب راحت، کنترل استرس، پیشگیری از وخامت بیماری، انجام فعالیت‌های روزانه، ورزش، رعایت رژیم غذایی سالم، بهره‌مندی از اوقات فراغت، جست‌وجوی معنی در زندگی و انجام فعالیت‌های ذهنی نشان داده شد. نتیجه‌گیری: رفتارهای شیوه زندگی سالم در سالمندان ایرانی متأثر از فرهنگ اسلامی-ایرانی و به دو دسته عمده رفتارهای متمرکز بر فرد و رفتارهای متمرکز بر جامعه قابل تقسیم است.

Title: تأثیر دوازده هفته تمرین در آب و خشکی روی تعادل ایستا و پویا و خطر سقوط افراد سالمند

http://salmandj.uswr.ac.ir/article-1-788-fa.html

اهداف: تحقیق حاضر، به‌منظور مقایسه تأثیر تمرین در آب و خشکی بر کنترل تعادل و خطر سقوط مردان سالمند سالم انجام شد. مواد و روش‌ها: بدین منظور تعداد 45 مرد سالمند بالای 65 سال به‌صورت تصادفی انتخاب و به سه گروه تمرین در آب، تمرین در خشکی و کنترل تقسیم شدند و هر گروه شامل 15 نفر بود. دو گروه تمرین در آب و گروه تمرین در خشکی به‌مدت12 هفته، هر هفته 3 جلسه و هر جلسه به‌مدت 60 دقیقه، در تمرینات مشابه در دو محیط آب و خشکی شرکت می‌کردند. یافته‌ها: یافته‌های تحقیق نشان داد که اعمال برنامه تمرین در آب و بیرون آب بر تعادل ایستا، پویا و خطر سقوط سالمندان تأثیر معنی‌داری دارد (P≤0/05). همچنین نتایج از تفاوت معنی‌داری بین داده‌های پس‌آزمون و آزمودنی‌های دو گروه تمرین و گروه کنترل حکایت دارد (P≤0/05)، هرچند تفاوت معنی‌داری بین نمرات پیش و پس‌آزمون گروه کنترل مشاهده نشد (P≥0/05). از سوی دیگر نتایج تفاوت معنی‌داری را بین دو گروه تمرین در آب و خشکی نشان نداد (P≥0/05). نتیجه‌گیری: براساس یافته‌های این پژوهش، به‌کارگیری تمرینات در آب و خشکی منجر به بهبود تعادل ایستا، پویا و خطر سقوط در سالمندان می‌شود.

Title: بررسی اپیدمیولوژی سقوط و ترس از سقوط در سالمندان و ارتباط آن‌ها در سرای سالمندی کامرانی تهران، سال 1392

http://salmandj.uswr.ac.ir/article-1-791-fa.html

اهداف: ترس از سقوط با محدودکردن فعالیت‌ها می‌تواند باعث افزایش خطر سقوط و آسیب شود. هدف از این مطالعه بررسی شیوع سقوط و ترس از سقوط در سالمندان و ارتباط آن‌ها با یکدیگر است. مواد و روش‌ها: این تحقیق از نوع توصیفی-تحلیلی به روش مقطعی است که در آن 160 فرد سالمند (130 زن و 30 مرد) با سن 65 سال به بالا با استفاده از نمونه‌گیری تمام‌شماری انتخاب شدند. پس از اخذ رضایت آگانه از فرم اطلاعات جمعیت‌شناختی، فرم ثبت دفعات سقوط و پرسش‌نامه خودکارآمدی ترس از سقوط برای جمع‌آوری داده‌ها استفاده شد. علاوه‌براین، از آمار توصیفی برای طبقه‌بندی داده‌ها و آمار استنباطی شامل تحلیل واریانس یک‌راهه، آزمون تی مستقل و همبستگی پیرسون برای تجزیه‌وتحلیل داده‌ها استفاده شد. ﺭﻭﺍﻳﻲ ﻭ ﭘﺎﻳﺎﻳﻲ پرسش‌نامه خودکارآمدی ترس از سقوط، پس از بررسی و تحلیل ﻣﻮﺭﺩ ﺗﺄﻳﻴﺪ ﻗﺮﺍﺭ ﮔﺮﻓﺖ (ﺁﻟﻔﺎﻱ ﻛﺮﻭﻧﺒﺎﺥ=%95). یافته‌ها: از بین 160 نمونه، بیشتر سالمندان زن (%81/2) و با میانگین (انحراف معیار) سنی 9/07±67/63 سال بودند. میانگین و انحراف معیار دفعات سقوط 0/85±2/6 بود. %46/9 از سالمندان دارای 3 بار سقوط، %33/8 دارای 2 بار سقوط و %9/4 دارای یک‌بار سقوط در شش ماه گذشته بودند. این در حالی است که بیشتر سالمندان (%76/2) از ترس از سقوط متوسط برخوردار بودند. بین جنس سالمندان و مصرف دارو با تعداد دفعات سقوط، تفاوت آماری معنی‌داری وجود داشت (0/001>P). همچنین بین ترس از سقوط و جنس (0/05>P) و سابقه اقامت (0/02=P) تفاوت آماری معنی داری مشاهده شد (0/001>P). نتایج حاصل از ضریب همبستگی پیرسون بین دفعات سقوط و ترس ازسقوط رابطه معناداری با اطمینان %99 نشان داد. نتیجه‌گیری: با توجه به شیوع سقوط و ترس از سقوط در سالمندان و تأثیر مستقیم آن‌ها بر یکدیگر، برنامه‌ریزی مناسب برای کنترل و پیشگیری از آنها به‌منظور افزایش رفاه سالمندان باید انجام گردد.

Title: بررسی شیوع سوء‌رفتار با سالمندان شهرستان‌های گرگان و آق‌قلا در سال 1392

http://salmandj.uswr.ac.ir/article-1-990-fa.html

اهداف: پدیده سالمندی و مسائل دنبال آن، به‌عنوان یکی از مهم‌ترین چالش‌های آینده در نظر گرفته می‌شود. مطالعات در زمینه سوءرفتار با سالمندان در ایران، بسیار محدود و ناچیز بوده است. پژوهش حاضر با هدف تعیین میزان و انواع سوءرفتار خانگی با سالمندان مراجعه‌کننده به مراکز بهداشتی شهرستان گرگان و آق‌قلا در سال 1393 انجام شده است. مواد و روش‌ها: این پژوهش توصیفی‌تحلیلی روی زنان و مردان سالمندی انجام گرفت که در مراکز بهداشتی شهری و روستایی شهرستان گرگان و آق‌قلا دارای پرونده بودند. در این مطالعه 465 نفر از این افراد به‌وسیله نمونه‌گیری تصادفی انتخاب شدند. جمع‌آوری اطلاعات از طریق مصاحبه چهره‌به‌چهره با سالمندان و تکمیل پرسش‌نامه «سوءرفتار با سالمندان»، پس از تأیید نبودِ مشکلات شناختی (کسب نمره 6 یا بالاتر از نسخه فارسی آزمون کوتاه وضعیت شناختی) انجام شد. داده‌ها با استفاده از نسخه 16 نرم‌افزار SPSS و با آمارهای توصیفی (درصد، فراوانی، میانگین و انحراف معیار) و آزمون‌های استنباطی (کای اسکوئر و آزمون تی) تحلیل گردید. یافته‌ها: یافته‌های پژوهش نشان داد که 63/3درصد از نمونه‌های پژوهش، حداقل یکی از انواع سوءرفتار را تجربه کرده بودند. بیشترین میزان شیوع سوءرفتار مربوط به غفلت مراقبتی (59/8درصد) و سپس سوءرفتار روان‌شناختی (53/3درصد) و کمترین آن مربوط به سوءرفتار جسمی (8درصد) و طردشدگی (8/2درصد) است. بین میزان سوءرفتار و متغیرهای جنس (0/034>P)، مکان زندگی (0001>P)، قومیت (0001>P) و سن (0/035>P) بین دو گروه سالمندان تحت سوءرفتار قرار گرفته و تحت سوء‌رفتار قرار نگرفته تفاوت معناداری وجود دارد. نتیجه‌گیری: با توجه به میزان بالای سوءرفتار نسبت به سالمندان، به نظر می‌رسد وجود برنامه‌هایی برای افزایش آگاهی سالمندان، مراقبان آن‌ها و پرسنل بهداشتی برای پیشگیری و کاهش غفلت و سوءرفتار با سالمندان ضروری باشد.

Title: کارآزمایی بالینی تأثیر مداخله مددکاری گروهی با رویکرد بخشش‌درمانی بر رضایت از زندگی سالمندان مقیم آسایشگاه

http://salmandj.uswr.ac.ir/article-1-983-fa.html

اهداف: رسیدن به سن سالمندی به‌همراه اقامت در آسایشگاه‌های سالمندان ممکن است منجر به کاهش رضایت از زندگی گردد. هدف از پژوهش حاضر تعیین اثرگذاری برگزاری جلسات گروهی بخشش‌درمانی بر رضایت از زندگی سالمندان مقیم آسایشگاه است.  مواد و روش‌ها: روش مطالعه حاضر از نوع مداخله شبه‌تجربی است. تعداد 55 نفر از چهار آسایشگاه نگهداری از سالمندان شهر تهران به‌صورت نمونه‌گیری تصادفی خوشه‌ای، انتخاب شدند. با توجه به ملاک‌های ورود، افراد مناسب شرکت در جلسات گروه درمانی انتخاب و افراد به‌صورت تصادفی به دو گروه مداخله و شاهد تقسیم شدند و پرسش‌نامه رضایت از زندگی روی ایشان تکمیل گردید و سپس گروه مداخله در هشت جلسه بخشش‌درمانی حضور یافتند و گروه شاهد مداخلات درمانی رایج را دریافت کردند. رضایت‌مندی از زندگی افراد پس از مداخله در دو گروه ارزیابی شد. ابزارهای جمع‌آوری شامل فرم مشخصات جمعیت‌شناختی و پرسش‌نامه رضایت از زندگی (LSI-Z)  (1969) بود و اطلاعات با استفاده از نسخه 16 نرم‌افزار SPSS تحلیل گردید. یافته‌ها: بین دو گروه در نمره رضایت از زندگی پیش از مداخله تفاوت معناداری مشاهده نشد، اما پس از مداخله به‌طوری‌که نتایج آزمون تی مستقل نشان داد، رضایت از زندگی گروه شرکت‌کننده در برنامه بخشش به‌طور معناداری نسبت به گروه شاهد افزایش داشته است و در مقایسه گروه مداخله و شاهد تفاوت معناداری مشاهده گردید (0/001>P). نتیجه‌گیری: شرکت در جلسات گروهی بخشش‌درمانی بر رضایت از زندگی سالمندان مقیم آسایشگاه تأثیرگذار است و انجام قراردادی برای افزایش بخشش، می‌تواند به ارتقای کیفیت زندگی سالمندان مقیم آسایشگاه کمک نماید.

Title: بررسی اعتبار و روایی آزمون زبان‌پریشی فارسی

http://salmandj.uswr.ac.ir/article-1-835-fa.html

اهداف: تاکنون «آزمون زبان‌پریشی فارسی» به‌عنوان نخستین و پرکاربردترین آزمون مدوّن برای سنجش و درمان اختلال‌های زبانی بیماران زبان‌پریش بزرگسال و تحقیقات زبان‌پریشی مورد استفاده قرار گرفته است. نسخه اولیه این آزمون براساس معیارهای انطباق زبانی و فرهنگی برای گویندگان سالم فارسی‌زبان معیاریابی و تدوین شد و همین نسخه افزون بر بیست سال است که در کلینیک‌های گفتاردرمانی مورد استفاده قرارگرفته است. هدف از مطالعه حاضر، بررسی روایی و اعتبار این آزمون براساس نتایج به‌دست‌آمده از بیماران زبان‌پریش فارسی‌زبان است. مواد و روش‌ها: در مطالعه حاضر، 57 بیمار بزرگسال فارسی‌زبان مبتلا به زبان‌پریشی (روان و ناروان) متعاقب ضایعه مغزی نیم‌کره چپ با دامنه سنی 19-83 سال به‌شیوه هدفمند در فاصله بین سال‌های 90-1385 از کلینیک‌های دانشگاهی و مراکز درمانی شهر تهران، مشهد، کرج، یزد، اصفهان و شیراز انتخاب شدند. برای بررسی روایی ملاکی آزمون، نیم‌رخ زبانی به‌دست‌آمده از نتایج این آزمون با تشخیص گفتاردرمانگر به‌منظور تعیین نوع زبان‌پریشی مقایسه و اعتبار آزمون از طریق محاسبه آلفای کرونباخ محاسبه شد. در ضمن برای بررسی روایی سازه آزمون، همبستگی بین مهارت‌های آزمون و خرده‌آزمون‌های تشکیل‌دهنده هر مهارت محاسبه شد. یافته‌ها: براساس نتایج این پژوهش، اعتبار کل آزمون 0/93 به‌دست آمد. از نظر ساختاری بین مهارت‌های آزمون، همبستگی معناداری وجود داشت (0/05>P و 0/76-0/03=R). همچنین خرده‌آزمون‌های هر مهارت از همبستگی متوسط تا بالایی برخوردار بودند(0/01>P و 0/83-0/63=R). از طرفی نتایج عملکرد انواع زبان‌پریشی در هر یک از مهارت‌های زبانی، نشان‌دهنده انطباق نیم‌رخ‌های به‌دست‌آمده با تشخیص گفتاردرمانگران بود. نتیجه‌گیری: با توجه به روایی و اعتبار بالای این آزمون، می‌توان از آن به‌عنوان یک ابزار بالینی معیار در تشخیص افتراقی انواع زبان‌پریشی و بررسی شدت آسیب‌پذیری مهارت‌های زبانی در مطالعات زبان‌شناسی بالینی در بیماران بزرگسال فارسی‌زبان دارای ضایعه مغزی استفاده کرد.

Title: بررسی ‌و مقایسه حیطه‌های مختلف سبک ‌زندگی سالم سالمندان ‌ساکن ‌خانه ‌سالمندان ‌شهر اصفهان

http://salmandj.uswr.ac.ir/article-1-820-fa.html

اهداف: جمعیت سالمندان جهان روز‌به‌روز در حال افزایش است و ایران نیز از این قاعده مستثنا نیست. با توجه به اینکه گسترده‌ترین علل مرگ در گروه‌های سنی بالای 50 سال با اصلاح سبک‌ زندگی قابل پیشگیری است، اتخاذ سبک‌ زندگی سالم در این دوران از اهمیت قابل توجهی برخوردار است. با توجه به این امر مطالعه حاضر به‌منظور بررسی وضعیت سبک ‌زندگی سالمندان ساکن در خانه سالمندان صورت گرفت. مواد و روش‌ها: این مطالعه توصیفی-تحلیلی از نوع مقطعی بود. جامعه پژوهش را 350 سالمند ساکن در خانه سالمندان شهر اصفهان تشکیل می‌دادند. اطلاعات به‌وسیله پرسش‌نامه استاندارد که مشتمل بر دو بخش جمعیت‌شناختی و ابعاد مختلف سبک ‌زندگی است، جمع‌آوری گردید. پس از تکمیل پرسش‌نامه‌ها توسط گروه هدف، داده‌ها با استفاده از نسخه 20 نرم‌افزار SPSS و آزمون‌های آماری توصیفی، همبستگی پیرسون، اسپیرمن و آزمون تی مستقل مورد تجزیه و تحلیل قرار گرفت. یافته‌ها: نتایج این مطالعه نشان داد که میانگین نمره سبک زندگی کل 8/71±49/81 است. علاوه‌براین، ضریب همبستگی پیرسون ارتباط معنی‌داری بین میانگین نمره سبک ‌زندگی کل با سطح تحصیلات (0/171=r و 0/001>P) و بیماری (0/009=P و 0/14-=r) نشان داد. نتایج آزمون همبستگی پیرسون حاکی از وجود ارتباط معنی‌دار بین ابعاد مختلف سبک ‌زندگی (0/05>P) به‌جز بُعد بهداشت روان و تغذیه بود. نتیجه‌گیری: با توجه به یافته‌های این پژوهش پیشنهاد می‌شود که سیاست‌گذاران سلامت، برای ارتقای وضعیت سبک ‌زندگی سالمندان ساکن در خانه سالمندان برنامه‌ریزی نمایند. با توجه به احساس تنهایی و انزوای سالمندان در این مراکز، به‌کارگیری راهبردهایی به‌منظور کاهش استرس و افسردگی و پیشگیری از آثار روانی و جسمانی آن، کاملاً ضروری به‌نظر می‌رسد. این امر می‌تواند گامی در جهت بهبود وضعیت سلامت این  قشر آسیب‌پذیر باشد.

Title: ادراک پیری در سالمندان: یک مطالعه مروری

http://salmandj.uswr.ac.ir/article-1-1011-fa.html

اهداف: ادراک پیری به‌عنوان یکی از پیش‌بینی‌کننده‌های مستقل ناتوانی عملکردی و مرگ‌ومیر سالمندان معرفی شده است. مطالعه حاضر با هدف بررسی مفهوم ادراک پیری در متون و مطالعات موجود و رسیدن به درک عمیق‌تری از آن انجام شد. مواد و روش‌ها: در این مطالعه دستیابی به اهداف با بررسی متون و مقالات مرتبط از طریق جستجو در پایگاه‌های اطلاعاتی «مِدلاین»، «مَگیران»، «ایران‌داک»، «اِلزِویر»، «پروکوییست سی.اس.اِی»، «سیناهِل»، «ایران‌مِدِکس» و «وب‌سایت علوم» و با کلیدواژگان انگلیسی Meaning of Ageing, Experience of Ageing, Ageing Perception in older Adult و کلیدواژگان فارسی «معنای پیری»، «تجربه سالمندشدن» و «درک از پیری»، بدون محدودیت زمانی انجام شد. یافته‌ها: درک از پیری به درک شخص از فرایند پیری خودش در زمینه فرهنگی-اجتماعی که قرار دارد، اطلاق می‌شود. تمام جنبه‌های آگاهی، درک، تجربه، ارزیابی، تفسیر و شناخت مراحل پیرشدن خود را می‌توان در این مفهوم جای داد. عوامل مؤثر بر ادراک پیری شامل عوامل فردی مانند نوع نگرش، سن ذهنی، وضعیت سلامتی، وضعیت اقتصادی، وضعیت تأهل، مذهب، آگاهی و دانش در زمینه سالمندی، میزان رضایت از پیری، میزان باور به کنترل درونی در قیاس با کنترل بیرونی و عوامل اجتماعی نظیر تبعیض سنی، نوگرایی (مدرنیته)، روابط اجتماعی و خانوادگی است. اندازه‌گیری دقیق ادراک پیری می‌تواند شاخصی برای پیش‌بینی وضعیت سلامتی فرد در آینده، تعیین رضایت‌مندی او از پیری خود و میزان تطابقش با تغییرات زندگی در این مرحله باشد. نتیجه‌گیری: مفهوم و عوامل مؤثر بر ادراک پیری در سالمندان، پیشگویی‌کننده‌ای مناسب برای وضعیت سلامتی، عامل تعیین‌کننده میزان رضایت‌مندی در دوران سالمندی و تطبیق با تغییرات مرتبط با پیری به‌شمار می‌آید و کندوکاو بیشتر در آن توصیه می‌شود .

Title: تحلیلی بر مشخصات روان‌سنجی ابزارهای سنجش حمایت اجتماعی درک‌شده در سالمندان

http://salmandj.uswr.ac.ir/article-1-1023-fa.html

اهداف: بخشی از ارزیابی وضعیت سلامتی سالمندان به سنجش حمایت اجتماعی درک‌شده اختصاص دارد. لازمه یک سنجش صحیح، استفاد‌ه از ابزارهایی مناسب با ویژگی‌های روان‌سنجی مطلوب است. هدف این مطالعه مروری، بررسی ویژگی‌های روان‌سنجی ابزارهای سنجش حمایت اجتماعی درک‌شده به‌منظور آشنایی محققان با نقاط قوت و ضعف این ابزارها و کمک به آنان برای استفاده از ابزاری با اعتبار بیشتر است. مواد و روش‌ها: برای انجام پژوهش حاضر، ابتدا جستجوی کاملی در بانک‌های اطلاعاتی «اسکوپوس»، «گوگل اسکالر» و «مِلاین» صورت گرفت و همه مقالات منتشرشد‌ه از سال 1970 تا پایان سال 2014 براساس کلیدواژه‌های مرتبط با طراحی و فرایند روان‌سنجی ابزارهای حمایت اجتماعی درک‌شد‌ه، مورد بررسی قرار گرفت. سپس با استفاد‌ه از چک‌لیست جامع کاسمین به بررسی ویژگی‌های روان‌سنجی ابزارها شامل روایی، پایایی، پاسخ‌گویی و تفسیرپذیری پرداخته شد.  یافته‌ها: بیشتر ابزارها ویژگی‌های روان‌سنجی کامل و مطلوبی را گزارش نکرد‌ه بود‌ند، برخی از ابزارها با وجود طراحی برای بالغان، به‌دلیل طولانی‌بودن ابزار، طولانی‌بودن سؤالات و وجود سؤالات خاص قابلیت کاربرد در جمعیت سالمند‌ان را نداشتند. نتیجه‌گیری: نتایج این مطالعه نشان داد که با وجود نبودِ ابزار مناسب برای سنجش حمایت اجتماعی درک‌شد‌ه سالمند‌ان، تنها ابزاری که می‌توان در حال حاضر استفاد‌ه از آن ‌را به سالمند‌شناسان توصیه نمود، ابزار پیمایش حمایت اجتماعی در مطالعه نتایج طبی است که در مطالعات مربوط به ارزیابی جامع سالمند‌ی به‌کاررفته است و لزوم طراحی ابزارهایی با ویژگی‌های سایکومتریک قابل قبول برای سنجش حمایت اجتماعی درک‌شد‌ه سالمند‌ان بر مبنای ویژگی‌های اجتماعی و فرهنگی جوامع مختلف احساس می‌شود.
